# Insights Into Opportunistic Lung Cancer Screening for Individuals Who Have Never Smoked

**DOI:** 10.1001/jamanetworkopen.2024.54009

**Published:** 2025-01-02

**Authors:** Roger Y. Kim

**Affiliations:** Division of Pulmonary, Allergy, and Care, Department of Medicine, University of Pennsylvania, Philadelphia.

Lung cancer remains the deadliest malignant neoplasm globally; and due to a combination of its high overall incidence and falling tobacco smoking rates, lung cancer among individuals who have never smoked (LCINS) now represents the seventh most common cancer and fifth leading cause of cancer-related deaths globally.^1^ Female sex, Asian ancestry, and family history of lung cancer are among the most frequently cited risk factors for LCINS, which overwhelmingly presents with adenocarcinoma histology, making it pathologically distinct from smoking-related lung cancer. As both the National Lung Screening Trial (NLST) and the Dutch-Belgian Randomized Lung Cancer Screening Trial demonstrated that lung cancer screening (LCS) with low-dose computed tomography (LDCT) reduces lung cancer mortality among individuals with a substantial smoking history, there has been considerable interest in evaluating LDCT for LCS among individuals who have never smoked (INS).^2^ To date, several high-profile, nonrandomized observational studies out of Asia have demonstrated the ability of LCS with LDCT to detect LCINS.^3,4^ However, the question of whether screen-detected LCINS predominantly represents early-stage lethal disease vs indolent nonlethal disease (ie, overdiagnosis) remains unanswered and an ongoing subject of debate.^4,5^

Among Asian INS, LCS with LDCT often detects subsolid (ie, pure ground-glass or part-solid) pulmonary nodules, thought to represent adenocarcinoma spectrum lesions which can range from atypical adenomatous hyperplasia to invasive adenocarcinoma.^6^ The salient knowledge gap in pathophysiology and key clinical management challenge is in predicting which lesions will eventually progress to invasive (ultimately fatal) disease and which will remain indolent throughout a patient’s lifetime. Taken together with the fact that the overall risk for lung cancer among INS is lower than that of those with a smoking history, routine LCS with LDCT for INS is not currently recommended.^2,5^ Despite this, in the setting of increased public awareness of LCINS, opportunistic LCS programs in East Asian countries such as China, Japan, and South Korea currently allow any individual—regardless of risk factors—to pay out-of-pocket to undergo LCS with LDCT.

In this multicenter cohort study of INS aged 50 to 80 years undergoing opportunistic LCS with LDCT at 2 hospital-affiliated health promotion centers across South Korea, Kim and colleagues^7^ sought to describe screen-detected lung cancer incidence and assess the association between sex and lung cancer outcomes. Individuals included in the study voluntarily received LCS between 2009 and 2021, and each had at least 12 months of follow-up data available. Those with an indeterminate pulmonary nodule identified via LDCT (ie, a positive screen) were referred to a pulmonary specialist for further workup. As this was a retrospective study of INS outside of routine LCS care, no standardized follow-up protocol existed; however, the investigators retrospectively categorized baseline LCS LDCT results using the American College of Radiology Lung Imaging Reporting and Data System (Lung-RADS) version 2022. Among the 21 062 individuals included in the study (76.6% women), 176 (0.8%) were diagnosed with LCINS as a result of LCS, with 141 (80.1%) manifesting as subsolid nodules on LDCT and 169 (96.0%) representing adenocarcinoma spectrum disease. LCINS detected in this study largely included premalignant or localized disease, with 164 (93.2%) diagnosed at either stage 0 (adenocarcinoma in situ) or stage I. The investigators did not identify any statistically significant differences by sex for any lung cancer outcomes and reported 5-year lung cancer-specific survival rates of 97.7% and 100% for women and men, respectively.

These findings by Kim et al^7^ highlight the potential risk for lung cancer overdiagnosis among INS undergoing indiscriminate LCS with LDCT, regardless of sex. The study builds upon a growing base of prior evidence suggesting screen-detected LCINS overdiagnosis. For example, a Taiwanese ecological cohort study demonstrated a precipitous increase in stage 0 and I lung cancer diagnoses and 5-year survival without a concomitant decrease in late-stage disease or lung cancer-specific mortality after implementation of opportunistic LCS.^4^ Data from the Taiwan Lung Cancer Screening for Never Smoker Trial (TALENT)^3^ similarly revealed a 96.5% stage 0 or I LCINS distribution, with 81.1% of cancers presenting as subsolid nodules. It is notable that compared with the 19.2% stage 0 LCINS reported by the TALENT investigators, the 2.8% stage 0 distribution documented by Kim and colleagues^7^ was considerably lower, underscoring the variability in downstream diagnostic evaluation intensity across different countries and health care systems. Strikingly, surgical lung biopsy was by far the most common initial invasive diagnostic procedure for pulmonary nodule evaluation in both the TALENT^3^ (93.2%) and Kim et al^7^ (83.5%) studies. When considering that surgical lung resection can be associated with mortality rates as high as 1% to 2%, the potential downstream harms associated with LCINS overdiagnosis cannot be overstated. While direct comparisons with the NLST are inherently challenging due to the lack of structured annual rounds of LCS in the Kim et al study,^7^ 94% of all baseline screens were negative (Lung-RADS 1 or 2 scan) in the Kim et al study^7^ compared with 86% in the NLST.^8^ Additionally, the false positive rate in the study by Kim and colleagues^7^ (proportion of individuals without lung cancer with a Lung-RADS 3 or 4 scan) was 5.5% compared with 12.8% in the NLST, providing further evidence of possible overdiagnosis.^8^

Extrapolating from these findings in Asian INS populations to apply to the US population is difficult due to differences in demographics, environmental factors, and clinical practice patterns for subsolid nodule management. To date, there are no active randomized clinical trials registered on ClinicalTrials.gov assessing LCS with LDCT for INS. However, there are several ongoing observational studies across the US to prospectively assess LCS among female Asian INS, including the University of California, San Francisco Female Asian Never Smokers (FANS) study and New York University Female Asian Nonsmoker Screening Study (FANSS). As the results from these studies and others are reported, it will be critical to interpret them within the context of prior Asian studies such as this one by Kim and colleagues.^7^ In the absence of high-quality randomized clinical trial data to determine the clinical efficacy of LCS for INS, the question of LCINS overdiagnosis will continue to loom.

This multicenter retrospective cohort study by Kim et al^7^ serves as another example of the challenges associated with implementing LCS for INS. It should serve as a cautionary tale when considering indiscriminate LCS for INS and further emphasizes the need to identify more selective, targeted criteria and randomized clinical trial evidence when evaluating the risks and benefits of LCS with LDCT in this patient population.
